# Pseudogenes as an alternative source of natural antisense transcripts

**DOI:** 10.1186/1471-2148-10-338

**Published:** 2010-11-03

**Authors:** Enrique M Muro, Miguel A Andrade-Navarro

**Affiliations:** 1 Max-Delbrück Center for Molecular Medicine. Robert Rössle Str, 10. 13125 Berlin, Germany

## Abstract

**Background:**

Naturally occurring antisense transcripts (NATs) are non-coding RNAs that may regulate the activity of sense transcripts to which they bind because of complementarity. NATs that are not located in the gene they regulate (trans-NATs) have better chances to evolve than cis-NATs, which is evident when the sense strand of the cis-NAT is part of a protein coding gene. However, the generation of a trans-NAT requires the formation of a relatively large region of complementarity to the gene it regulates.

**Results:**

Pseudogene formation may be one evolutionary mechanism that generates trans-NATs to the parental gene. For example, this could occur if the parental gene is regulated by a cis-NAT that is copied as a trans-NAT in the pseudogene. To support this we identified human pseudogenes with a trans-NAT to the parental gene in their antisense strand by analysis of the database of expressed sequence tags (ESTs). We found that the mutations that appeared in these trans-NATs after the pseudogene formation do not show the flat distribution that would be expected in a non functional transcript. Instead, we found higher similarity to the parental gene in a region nearby the 3' end of the trans-NATs.

**Conclusions:**

Our results do not imply a functional relation of the trans-NAT arising from pseudogenes over their respective parental genes but add evidence for it and stress the importance of duplication mechanisms of genetic material in the generation of non-coding RNAs. We also provide a plausible explanation for the large transcripts that can be found in the antisense strand of some pseudogenes.

## Background

Non-coding RNA transcripts have emerged as an important type of regulatory molecules [1,2], in particular, Natural Antisense Transcripts (NATs) that can bind by partial complementarity to sense RNA transcripts to modulate their processing [3,4]. Their generation and mechanism of action are different to those of miRNAs, which are processed into shorter 21 nt products and have possibly less specific effects [5].

Complementarity to target transcript, which is a requirement for a NAT to have an effect, is evident if the NAT is expressed in cis to the sense transcript (that is, the NAT is located in the antisense strand of its target sense transcript), but this ties the evolution of both the sense transcript target and its cis-NAT [6].

Trans-NATs, on the other hand, are transcribed from a sequence that it is not located in the same genomic locus of their target and can then evolve separately constrained only by keeping a complementary region to the target gene [7]. Against accumulating evidence about trans-NATs, the puzzle remains of how relatively large and specific complementary regions can arise to form such anti-sense transcripts. A possibility that we raise here is that given a parental gene regulated by a cis-NAT, the duplication of the genomic fragment including the cis-NAT may result in a pseudogene holding an active copy of the cis-NAT, which is naturally a trans-NAT of the parental gene. Then, evolution can eliminate any of the NATs or tune their expression differently. More generally, the formation of any pseudogene results in complementary regions to the parental gene which, if combined with elements of transcriptional control antisense to the pseudogene, can conceivably lead to the generation of a trans-NAT antisense the pseudogene that is a potential regulator of the parental gene.

Antisense transcription from pseudogenes in mammals was discovered in human [8] and mouse [9], but with unknown function. Although it has been estimated that up to 20% of human pseudogenes can originate transcripts [10] there is little evidence of such transcripts having an effect in the expression of the parental genes or any other functionality for that matter [11]. Possibly, the best characterized example is the neuronal nitric oxide synthase gene (nNOS) in the central nervous system of the snail *Lymnaea stagnalis*. In this case, the nNOS pseudogene is itself a trans-NAT that inhibits the expression of nNOS [12], and this trans-NAT is expressed in a conditioning-dependent manner indicating a role in learning and long-term memory [13]. Other less-direct evidence of transcription from pseudogenes with an effect on the expression of their parental genes was given by the finding of pseudogenes that are the source of dsRNAs regulating gene expression in mouse oocytes [14,15]. Expression of possible siRNAs from pseudogenes has also been studied in rice [16].

An explanation for this small number of cases could be that the evolution of a functional trans-NAT from the antisense strand of a pseudogene eventually erases the traces of the pseudogene; therefore, the possibility of observing a trans-NAT related to a pseudogene could be transitory in evolutionary terms. For this reason, an exhaustive study of the database of transcripts to find and analyze the expression and sequence of antisense transcripts from pseudogenes is necessary to show evidence of these mechanisms.

We identified pseudogenes with transcription from their antisense strand; by definition, these are complementary to the parental gene of the pseudogene and can be defined as trans-NATs of that parental gene. Then, we studied the alignment between the DNA sequence the trans-NAT is transcribed from and the parental gene. In particular, we obtained the distribution of the mutations that appeared in the trans-NAT after the pseudogene formation. We observed distinctly a higher similarity between the sequences in a 50 nt region nearby the 3' end of the antisense transcript. This shows an increased selection pressure to keep the similarity between the pseudogene and the parental gene in the region that corresponds to the end of the trans-NAT and suggests a functional association between the trans-NATs and the parental gene.

## Results

We found 87 transcripts expressed antisense of human pseudogenes by analysis of the expressed sequence tag (EST) database (see Methods section). These transcripts are complementary to the parental gene and are by definition trans-NATs. The ESTs used as evidence were selected to align significantly better to the pseudogene than to any other genomic location. The direction of their transcription was verified using as evidence the strongest Poly-Adenylation Signals (PAS: "AAUAAA" and "AUUAAA") found within 30 nt of their 3'-end [17], splicing signals and cDNA-end poly-A tracts.

**Figure 1:**
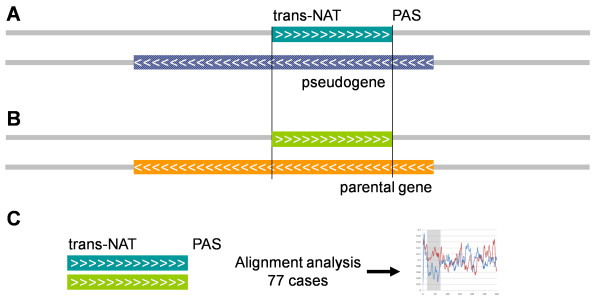
**Procedure of analysis followed in this study**. (A) Selection of transcripts with PAS and other evidence for transcription (trans-NAT, dark green box; see Methods) in antisense of pseudogenes (hatched box). (B) Next, the corresponding region in the antisense of the parental gene (light green box) is obtained. (C) The alignments of 77 selected trans-NATs to their corresponding regions in the antisense parental genes were used to study their identity levels as represented in Figure 2 below.

We then aligned the trans-NAT to its homologous region in the antisense strand of the parental gene (see Figure 1). The goal is to study the position of the mutations that appeared in the trans-NAT region after the pseudogene formation. In most cases (77 cases out of 87, see Methods) the alignment on the parental gene included the region that surrounds the PAS of the trans-NAT. In 15 cases we found ESTs in the parental gene region that suggested the expression of a cis-NAT equivalent to the observed trans-NAT.

**Figure 2:**
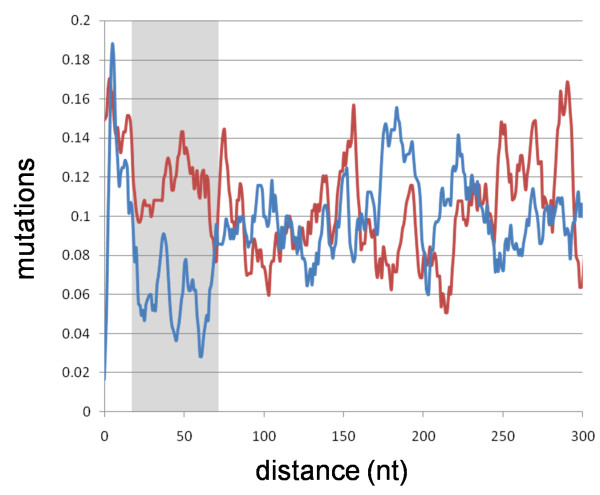
**Fraction of 77 pseudogenes (putatively expressing an EST in anti-sense) that have a mutation respect to the homologous position in the parental gene at a given position in their sequence**. Blue curve: distance is taken upstream the putative PAS of the EST +10 nt (the PAS is at position × = 10); the curve indicates that there is a region of high conservation in a 50 nt region upstream the putative PAS (gray box). Red curve: distance is taken downstream the 5'-end of the EST -100 nt; no region of high conservation is present. Values are averaged in a window of five nucleotides. The difference between the two distributions is statistically significant (p-value = 4.482 × 10^-7 from a Kolmogorov-Smirnov test).

To study the patterns of evolution of the 77 trans-NATs that fully aligned to the parental gene, we generated a histogram that describes the sequence identity between the pseudogene region originating the trans-NAT and the parental gene (Figure 2). Neutral evolution would be indicated by a flat distribution of identity, whereas variations from this distribution should show selection pressure for some sequence features that would point to functionality [18]. Most of the contribution to this observation is due to a lower occurrence of indels (Additional file 1: figure S1).

The plot shows a region of higher identity extending about 50 nt upstream the PAS of the trans-NATs expressed antisense to pseudogenes. This implies that a lower than expected level of mutations appeared in a region nearby the 3' end of the trans-NATs after the pseudogene formation Such selection pressure to preserve a region of complementarity to the parental gene suggests that these transcripts may be functioning as regulators of the parental gene. These results are not too sensitive to the quality of the alignment between EST and pseudogene (See Additional file 2: Figure S2).

In the next paragraphs we show some examples of the trans-NATs expressed in antisense to pseudogenes found in this study in more detail. Details about these examples and about the complete set are in Additional file 3 and 4: Tables S1 and S2.

### A trans-NAT expressed antisense of a pseudogene for a parental gene without cis-NAT evidence

**Figure 3:**
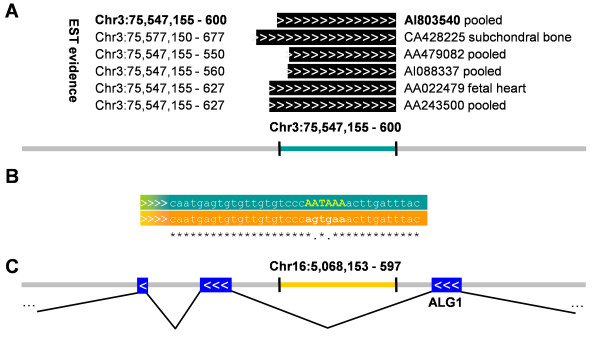
**A trans-NAT expressed antisense of a pseudogene**. (A) Genomic features in a region of human chromosome 3 (negative strand, coordinates decrease from left to right). EST AI803540 is expressed in this region from positions 75,547,155 to 75,547,600 and represents a trans-NAT expressed antisense of a pseudogene. Another five ESTs have an identical or very close 3'-end position and support the same antisense transcript. "Pooled" indicates that the EST was obtained from pooled human melanocyte, fetal heart, and pregnant uterus. (B) This genomic region is highly identical to the parental gene region in Chromosome 16, from positions 5,068,153 to 5,068,597, as indicated by sequence alignment (partially represented in the figure). The PAS of the trans-NAT ("AATAAA", in yellow) is not conserved in the parental gene. (C) The region of similarity is located in an intron of gene *ALG1 *and it is antisense of the direction of transcription. No evidence of antisense transcription was found in this region of the parental gene and some mutations happened in the positions aligning to the PAS of the trans-NAT.

AI803540 (446 nt, from a library pooled from human melanocyte, fetal heart, and pregnant uterus) aligns antisense to a pseudogene (chromosome 3, 75,547,155 - 75,547,600; UCSC hg18 genome version equivalent to NCBI Build 36.1) and has a PAS "AATAAA" at position 75,547,171 (Figure 3). The region of the pseudogene originating this trans-NAT aligns to the 6^th ^intron of the parental gene (*ALG1*) in chromosome 16 (5,068,153 - 5,068,597). The identity between the pseudogene and the parental gene regions is 95% all over the 446 nt of the EST, but the parental gene lacks the equivalent PAS due to two mutations ("AGTGAA").

In agreement to this, there is no cDNA evidence of the expression of a cis-NAT in the corresponding region of *ALG1*. We propose that EST AI803540 represents a trans-NAT that could interact with the pre-mRNA of *ALG1*. Another 5 ESTs support AI803540. *ALG1 *encodes a protein glycosyl transferase that was associated to a severe congenital disorder of glycosylation producing death in early infancy [19].

### A trans-NAT expressed antisense of a pseudogene for a parental gene with a corresponding cis-NAT

AA897638 (400 nt, from a library pooled from fetal lung, testis, and B-cells) aligns antisense to a pseudogene (chromosome 9, 87,585,882 - 87,586,276) and has a PAS at position 87,585,900. The corresponding pseudogene region is 98.5% identical to a region less than 70 Kb away (85,646,797 - 85,647,191) that includes an exon from gene *KIF27 *(encoding kinesin family member 27) and flanking regions. Additional EST evidence supports the expression of both the trans-NAT from the pseudogene and of a cis-NAT from the parental gene using the equivalent antisense PAS (with 2 and 4 ESTs, respectively). *KIF27 *is a homolog of *Drosophila melanogaster *Costal-2, and as such it is expected to participate in the Hedgehog signaling pathway, but its activity has not been yet experimentally studied.

### A trans-NAT expressed antisense of a pseudogene whose 3'-end region does not align to the parental gene

AA906308 (277 nt, from a library pooled from fetal lung, testis, and B-cells) aligns antisense the pseudogene to chromosome 17 (22,116,782 - 22,117,058) with PAS "AATAAA" at position 22,117,040. Another 3 ESTs support this trans-NAT 3' end. It is expressed in antisense of a processed pseudogene of gene *WEE1*, and aligns (with > 90% identity) to exons 9 and 10 of this gene in chromosome 11 (9,564,608 - 9,564,869) but not to the intron spanning them. The *WEE1 *gene encodes the wee1 tyrosine kinase [20], a key G2 phase cell cycle regulator. AA906308 aligns well to the *WEE1 *gene except for its 3'-end. In agreement to this, no evidence of cis-NAT expression was found in the parental gene.

Comparisons to other organisms show that this processed pseudogene is also present in chimpanzee (in this case with two copies in chromosome 17) and orangutan (three copies in chromosome 17), but not in the rhesus macaque (*Macaca mulatta*). Therefore, it seems that there is selection pressure to generate copies of this pseudogene in the Hominoidea lineage, and trans-NATs to *WEE1 *may exist in other organisms, possibly in multiple copies. The next example, illustrates a case of multiplicity of antisense trans-NATs from multiple pseudogene copies with more abundant evidence.

### An ensemble of trans-NATs expressed antisense of multiple pseudogene copies

Trans-NATs expressed antisense of pseudogenes can be duplicated through events of genomic duplication. We illustrate this with EST AA149869, located in chromosome 17 (42,479,675 - 42,480,254), which represents a trans-NAT with support from another 15 ESTs from different tissues (adult eye, fetal, and glioblastoma) that terminate at the same PAS. AA149869 is highly complementary to nine regions in chromosome 17, five of them in antisense to introns of four homologous protein coding genes of uncharacterized function: (LRRC37A3, LRRC37A2, LRRC37A and LRRC37B) and one pseudogene (LRRC37B2). Therefore this transcript could potentially regulate four genes. Three of those have evidence of expression of the corresponding antisense cis-NAT. Further EST evidence suggests an additional trans-NAT in chromosome 17 with homology to EST AA149869. The possible regulatory interactions between such a matrix of four human genes, three cis-NATs and two trans-NATs seems complex.

Examination of the genomes of other organisms shows the existence of equivalents of these pseudogenes in chromosome 17 of both the chimpanzee and the orangutan, and in chromosome 16 of the rhesus macaque (*Macaca mulatta*), and no significant similarity in other organisms such as the marmoset (a primate) or rodents (mouse, rat, and guinea pig) (sequences are available as Additional file 5). The phylogenetic analysis of the pseudogene sequences (not shown) suggests multiple independent replications of this pseudogene along the Catarrhini lineage.

## Discussion

We have collected evidence of the expression of transcripts antisense of pseudogenes, which would be trans-NATs of the corresponding parental genes. Some of these transcripts are supported by one single EST and we do not expect that all transcripts collected will represent true transcripts. However, even though our collection may contain false positives, when considered collectively our study indicates that these trans-NAT sequences have higher similarity to their parental genes in the region 50 nt upstream their 3' ends. This similarity is distinctively higher than the sequence identity between pseudogenes and parental genes observed further upstream that region (Figure 2). This suggests that many of these transcripts are under selective pressure, evidenced by a mutation rate in that region lower than in other parts of the pseudogene; one possible interpretation of this observation is that many of these trans-NATs are expressed and therefore that pseudogene formation results in the generation of trans-NATs that could be functional.

Some cases where cis-NAT evidence was found in the parental gene suggest that a trans-NAT can result from the pseudogenization of a gene with an already existing cis-NAT; we found 15 cases where EST evidence shows that such transcript antisense the parental gene is expressed. On the contrary, in 17 of the cases analysed, mutations of the corresponding PAS in the parental gene suggest that further evolution led to the inactivation of an original cis-NAT while the trans-NAT in the pseudogene was maintained (Figure 3).

Pseudogene trans-NATs that arise by genetic duplication of entire cis-NATs likely conserve surrounding cis-regulatory motifs that control their epigenetic regulation and their specific expression. Evidence in this direction is given by the observation that in our selection of pseudogenes with trans-NATs, the fraction of duplicated pseudogenes is dominant, as opposed to what was observed for the total set of human pseudogenes we used as source (See Table 1): formation of a duplicated pseudogene includes the introns of the gene and is more likely to include both the cis-NAT and its surrounding control regions.

**Table 1:** Percentage of pseudogenes by type

	**duplicated**	**ambiguous**	**processed**
	
Total^1^	10%	39%	51%
With antisense transcript^2^	80%	5%	15%

^1^The percentage of pseudogenes by type was obtained from the pseudogene.org database.

^2^From the subset of those where we detected trans-NAT expression.

Ten trans-NATs expressed antisense of pseudogenes that lack sequence similarity in their 3'-end to the parental gene suggest an alternative mechanism of pseudogene trans-NAT production. The example presented (an antisense transcribed from a processed pseudogene of gene *WEE1*) has levels of above 90% identity to the sequence antisense of two consecutive exons of the parental gene spanning more than 150 nt. The region of the pseudogene corresponding to the 3' end of this trans-NAT has no significant similarity to the parental gene. One possibility is that there was an original cis-NAT in the parental gene whose 3'-end was deleted after the production of the pseudogene and the subsequent evolution of the trans-NAT. Other possibilities are that the trans-NAT was formed by the insertion of the processed pseudogene on an existing transcription unit or that the trans-NAT regulatory regions arose de novo for the pseudogene. At this point, we cannot provide evidence for any of these possibilities.

We have presented examples showing selective pressure acting along the Hominoidea lineage for the duplication of genes and their cis-NATs and trans-NATs in particular chromosomal regions. Such may result in ensembles of genes commonly regulated by groups of NATs generated in their vicinity. Several such regions with a high rate of local duplications have been described and their evolution among primates is under study but it is not yet clear whether they are accidents of evolution or confer a selective advantage [21].

## Conclusions

Our observations support pseudogene formation as a mechanism of functional trans-NAT generation. Our set of examples adds evidence for the importance of duplication mechanisms of genetic material for the generation of non-coding RNAs and gives a plausible explanation for the generation of relatively large complementary transcripts like trans-NATs.

## Methods

The genome-wide (Build NCBI 36.1) set of human pseudogenes was obtained from (http://www.pseudogene.org/ downloaded on 22 Feb 2008) [22]. This version of the database contained 20,625 pseudogenes. Of those, 20,197 were associated to a parental gene annotated with an Ensembl gene id http://www.ensembl.org. Of those, 2,022 (10%) were unprocessed pseudogenes, 10,346 (51%) were processed and 7,829 (39%) were ambiguous (see Table 1). A total of 4,484 different parental genes were obtained that had a valid Ensembl gene identifier and a unique location in autosomes 1-22 and chromosomes × and Y.

The EST libraries offer a resource to study the expression of hundreds of thousands of transcripts. It is possible to deduce their relevance and cleavage by analysis of redundant EST sequences and of genomic PAS [17]. EST cDNA libraries are produced using priming to the poly-A tail of the transcript and therefore ESTs will generally not represent the totality of the transcript but its 3' end, up to around 800 nt.

To search for ESTs representing transcripts antisense to pseudogenes, we selected ESTs from the GenBank database (through the UCSC Genome Browser; http://genome.ucsc.edu/) that aligned to any one of the pseudogenes as deduced by alignment (BLAT score/qSize > = 0.90 and pid > = 90%) of the EST to the genome. This avoids the need to consider polyA or RNA editing.

We then selected ESTs antisense to the pseudogene. The sense of the EST was evaluated by the presence of one of the two strong PAS signals ("AAUAAA" or "AUUAAA") within 30 nt of the end of the transcript [17], splicing signals and cDNA end poly-A tracts. For the sake of confidence, the PAS is identified in the EST and also in the antisense sequence of the pseudogene. A total of 1,044 ESTs where selected using these conditions.

In order to make sure that the genomic origin of the ESTs is from the pseudogene, and not from the parental gene or any other genomic location, we discarded those that had multiple alignments to the genome according to the UCSC criteria (with an alignment having a base identity level within 0.5% of the best and at least 96% base identity with the genomic sequence). We preserved 349 ESTs. Three further ESTs were eliminated because the pseudogene overlapped with its parental gene.

We clustered the 346 transcripts according to their PAS into 182 groups of ESTs ending in the same PAS. These groups originated from 116 different pseudogenes corresponding to 103 different parental genes. The EST in each of the 182 clusters with the best alignment to the pseudogene (according to UCSC Genome Browser's sorting of BLAT results) was chosen as representative.

We aligned the corresponding genomic sequences of the 182 representative ESTs to their corresponding parental genes using BLAT, and excluded all those that did not align significantly. We ended up with 87 trans-NATs located in 61 pseudogenes related to 58 parental genes (see Additional file 3 and 4: Tables S1 and S2). Of the pseudogenes, 80% were unprocessed, 15% were processed and 5% were ambiguous (see Table 1). For the analysis of the distribution of mutations along pseudogenes presented in figure 2 we excluded the 10 cases for which the region that surrounded the PAS in the pseudogene did not align to the parental gene. These cases are indicated with a 1 in the column 10 of the Additional file 3: Table S1 and their alignments to the parental can be seen in the Additional file 4: Table S2. In addition, since we have focused the analysis on the description of the mutations in the sequence of the pseudogenes respect to the parental genes, insertions in the parental gene were not considered.

## Authors' contributions

EM and MA conceived and designed the experiments, analyzed the data and wrote the paper. EM performed the experiments. All authors read and approved the final manuscript.

## Supplementary Material

Additional file 1 **Figure S1. Number of gaps studied in **Figure 2. Number of gaps in 77 pseudogenes (putatively expressing an EST in anti-sense) studied in Figure 2. Blue curve: distance is taken upstream the putative PAS of the EST +10 nt (the PAS is at position × = 10). Red curve: distance is taken downstream the 5'-end of the EST -100 nt; no region of high conservation is present. Values are averaged in a window of five nucleotides. Both curves follow closely the ones shown in Figure 2 suggesting that most of the contribution to the effect described is due to the absence of gaps in the region around 50 nt upstream the antisense PAS. Click here for file

Additional file 2 **Figure S2. Mutations within the 62 trans-NAT with the best alignments**. Fraction of 62 pseudogenes (putatively expressing an EST in anti-sense) that have a mutation respect to the homologous position in the parental gene at a given position in their sequence. This is the subset of the 77 pseudogenes represented in Figure 2 with alignments of highest quality (> = 97% of the length of the EST is mapped onto the genome) to their representative ESTs. Blue curve: distance is taken upstream the putative PAS of the EST +10 nt (the PAS is at position × = 10). Red curve: distance is taken downstream the 5'-end of the EST -100 nt; no region of high conservation is present. Values are averaged in a window of five nucleotides. Click here for file

Additional file 3 **Table S1. List of transcripts expressed antisense of pseudogenes**. The columns indicate: (1) position of the parental gene with Ensembl gene identifier, chromosome, strand, start and stop position; (2) position of the pseudogene with pseudogene identifier, chromosome, strand, start and stop position; (3) type of pseudogene; (4) position of representative EST of antisense transcript with EST identifier, chromosome, strand, start and stop position; (5) position of the PAS of the representative EST; (6) library (7) developmental stage and (8) tissue of the representative EST; (9) equivalent antisense PAS conserved in the parental gene: 1 = yes, 2 = no; (10) the region surrounding the PAS of the representative EST does not align to the parental gene: 1 = true; 0 = false; (11) identifiers of other ESTs in the cluster. Click here for file

Additional file 4 **Table S2. EST and genomic evidence of 87 transcripts expressed antisense of pseudogenes**. There are five rows for each trans-NAT. The first row (starting with " > ") contains 13 fields: (1) gene location; (2) pseudogene location; (3) pseudogene type; (4) position of polyadenylation signal; (5) number of ESTs supporting the antisense trans-NAT; (6) identifier of representative EST; (7) BLAT score and (8) BLAT percentage of identity of the alignment between the EST and the parental gene; (9) equivalent antisense PAS conserved in the parental gene: 1 = yes, 2 = no; (10) number of ESTs supporting the antisense cis-NAT; (11) length, (12) number of point mutations and (13) gaps in the alignment between pseudogene and parental gene. The second row shows the sequence of the representative EST. Rows 3 to 5 show the alignment of the pseudogene region originating the EST with the parental gene. Click here for file

Additional file 5 **Sequences of 10 human and 18 primate homologous pseudogenes**. The sequences are in a FASTA file format. Sequence identifiers start with a code for the species name (hs = *Homo sapiens*, ch = chimpanzee, or = orangutan, rh = rhesus) followed by the start and stop positions and the direction (plus or minus) of the sequence. The name is followed by the scores output from a UCSC BLAT search of the sequence against the corresponding genome and some comments in the case of the human sequences regarding the position of the sequence respect to protein coding genes and the existence of EST evidence or lack of it. Click here for file
