# Supplementary figures and images for: Pseudogenes as an alternative source of natural antisense transcripts
Source: BMC Evol Biol. 2010 Nov 3;10:338. doi: 10.1186/1471-2148-10-338 (PMC2984423; doi:10.1186/1471-2148-10-338)

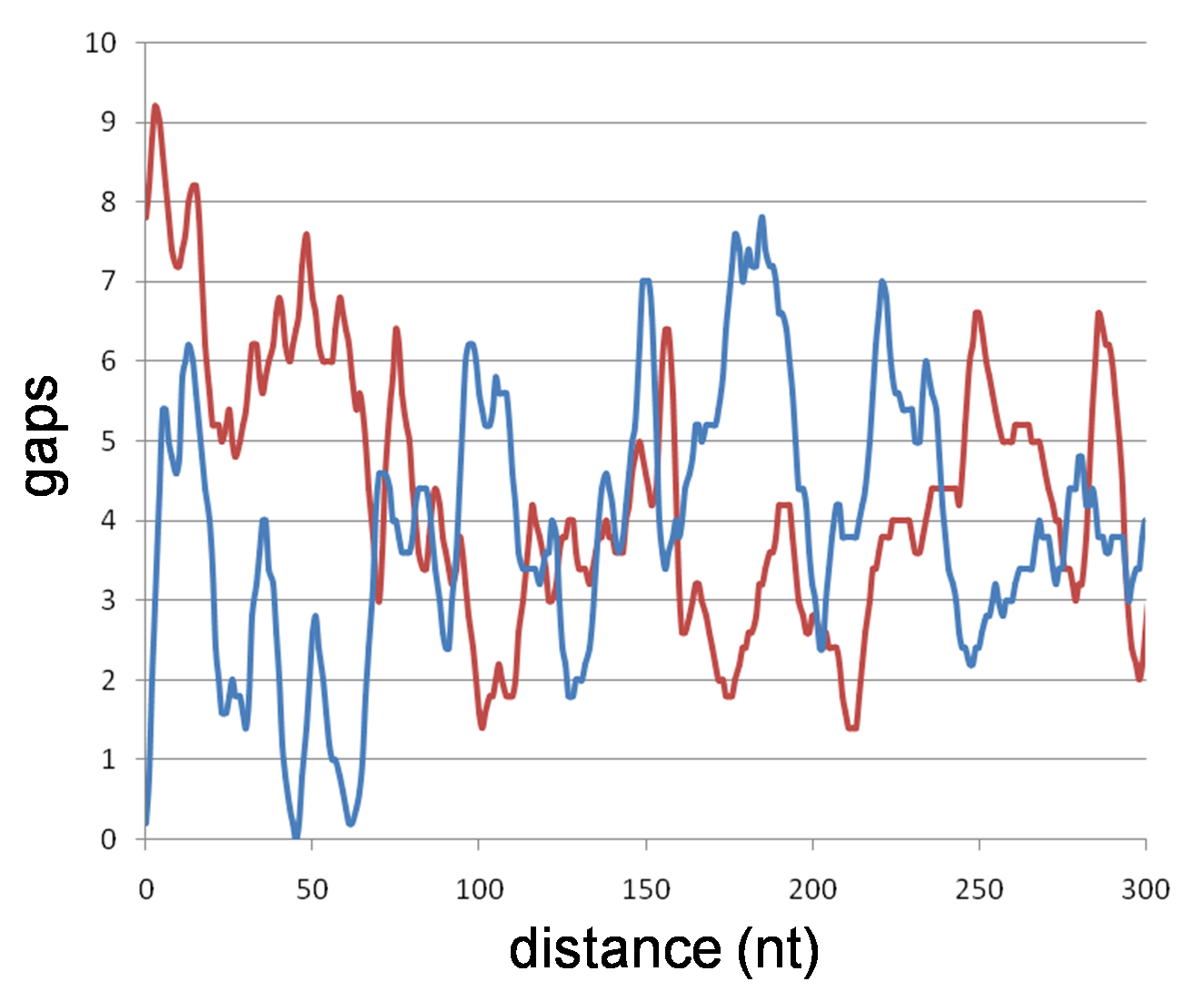

Supplement: Additional file 1 — Figure S1. Number of gaps studied in Figure 2. Number of gaps in 77 pseudogenes (putatively expressing an EST in anti-sense) studied in Figure 2. Blue curve: distance is taken upstream the putative PAS of the EST +10 nt (the PAS is at position × = 10). Red curve: distance is taken downstream the 5'-end of the EST -100 nt; no region of high conservation is present. Values are averaged in a window of five nucleotides. Both curves follow closely the ones shown in Figure 2 suggesting that most of the contribution to the effect described is due to the absence of gaps in the region around 50 nt upstream the antisense PAS. [file 1471-2148-10-338-S1.TIFF]

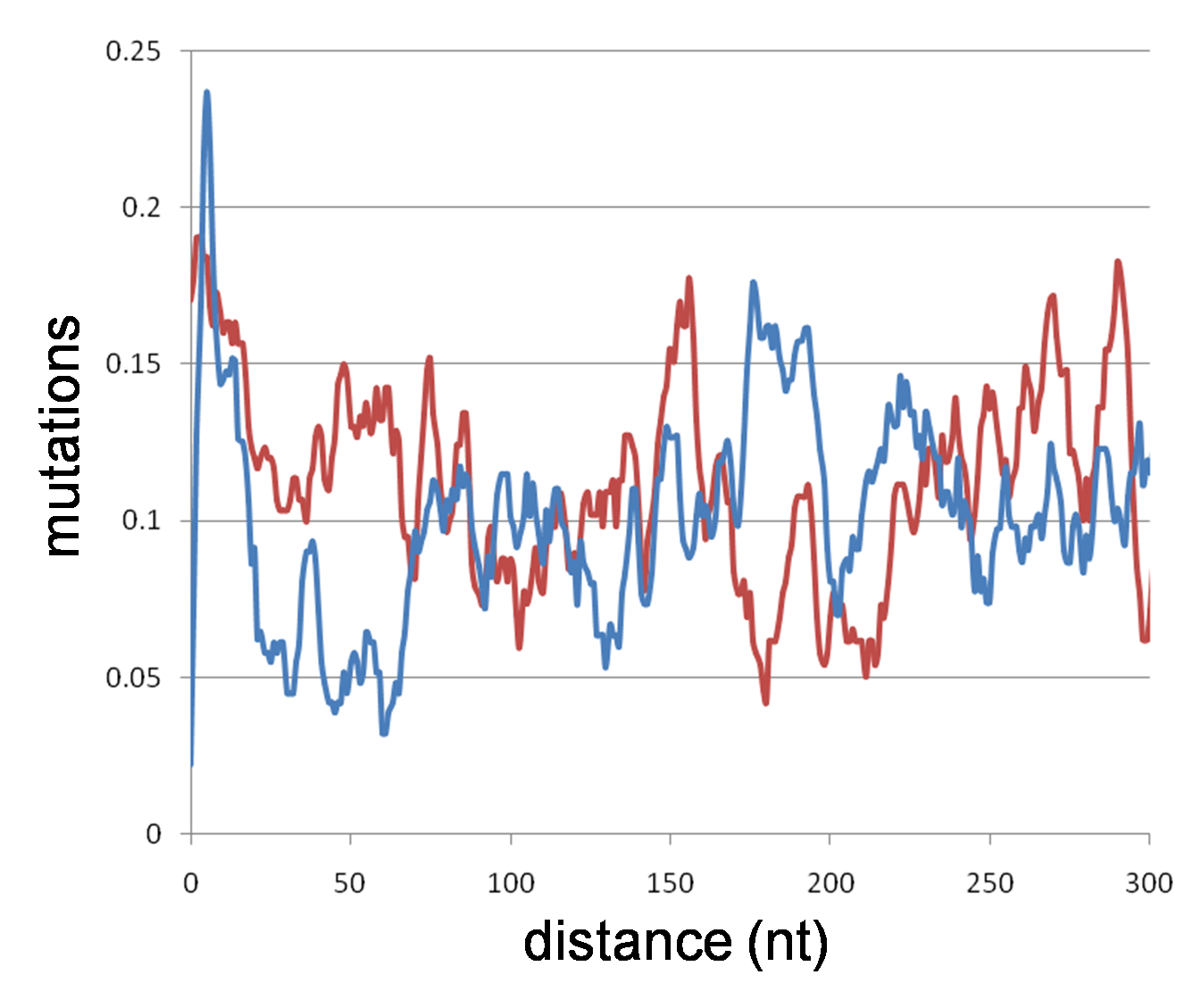

Supplement: Additional file 2 — Figure S2. Mutations within the 62 trans-NAT with the best alignments. Fraction of 62 pseudogenes (putatively expressing an EST in anti-sense) that have a mutation respect to the homologous position in the parental gene at a given position in their sequence. This is the subset of the 77 pseudogenes represented in Figure 2 with alignments of highest quality (> = 97% of the length of the EST is mapped onto the genome) to their representative ESTs. Blue curve: distance is taken upstream the putative PAS of the EST +10 nt (the PAS is at position × = 10). Red curve: distance is taken downstream the 5'-end of the EST -100 nt; no region of high conservation is present. Values are averaged in a window of five nucleotides. [file 1471-2148-10-338-S2.TIFF]
